# IMPATT Diodes Based on 〈111〉, 〈100〉, and 〈110〉 Oriented GaAs: A Comparative Study to Search the Best Orientation for Millimeter-Wave Atmospheric Windows

**DOI:** 10.1155/2015/484768

**Published:** 2015-03-10

**Authors:** Bhadrani Banerjee, Anvita Tripathi, Adrija Das, Kumari Alka Singh, Aritra Acharyya, J. P. Banerjee

**Affiliations:** ^1^Supreme Knowledge Foundation Group of Institutions, Mankundu, Hooghly, West Bengal 712139, India; ^2^Institute of Radio Physics and Electronics, University of Calcutta, 92 APC Road, Kolkata 700009, India

## Abstract

The authors have carried out the large-signal (L-S) simulation of double-drift region (DDR) impact avalanche transit time (IMPATT) diodes based on 〈111〉, 〈100〉, and 〈110〉 oriented GaAs. A nonsinusoidal voltage excited (NSVE) L-S simulation technique is used to investigate both the static and L-S performance of the above-mentioned devices designed to operate at millimeter-wave (mm-wave) atmospheric window frequencies, such as 35, 94, 140, and 220 GHz. Results show that 〈111〉 oriented GaAs diodes are capable of delivering maximum RF power with highest DC to RF conversion efficiency up to 94 GHz; however, the L-S performance of 〈110〉 oriented GaAs diodes exceeds their other counterparts while the frequency of operation increases above 94 GHz. The results presented in this paper will be helpful for the future experimentalists to choose the GaAs substrate of appropriate orientation to fabricate DDR GaAs IMPATT diodes at mm-wave frequencies.

## 1. Introduction

Impact avalanche transit time (IMPATT) diodes are well recognized two terminal solid-state devices to deliver sufficiently high power at both microwave and mm-wave frequency bands [1]. Silicon is the most popular base material for IMPATT diodes from the point of view of its advanced process technology [2–6]. However, GaAs is another vital base semiconductor for IMPATT diodes at the both microwave and mm-wave frequencies. Since early seventies, several researchers have fabricated IMPATT diodes based on GaAs and obtained higher DC to RF conversion efficiency and better avalanche noise performance of those as compared to their conventional Si counterparts [7–11].

The carrier ionization rates in a semiconductor material are key parameters which govern the RF performance of IMPATT sources. The inequality in ionization rates of electrons and holes (i.e., *α*_*n*_ ≠ *α*_*p*_) in GaAs was first reported in late seventies [12]. Pearsall et al. [13] experimentally measured the carrier ionization rates in GaAs under the electric field along the normal to 〈111〉, 〈100〉, and 〈110〉 oriented crystal substances. They reported different values of *α*_*n*_ and *α*_*p*_ for different orientations. Thus, it is evident from the above-mentioned report [13] that the carrier ionization rates in GaAs depend significantly on the crystal orientation of the substrate. Since the RF performance of IMPATT diode is strongly dependent on the carrier ionization rates of the base material, the same must be significantly influenced by the crystal orientation of the substrate (here GaAs). This fact encouraged the authors to carry out a comparative study on the L-S performance of DDR IMPATT diodes based on 〈111〉, 〈100〉, and 〈110〉 oriented GaAs. Earlier in 1993, Pati et al. [14] investigated the high frequency properties of 〈111〉, 〈100〉, and 〈110〉 oriented *p* ^+^-*n*-*n* ^+^, *n* ^+^-*p*-*p* ^+^ (single-drift region (SDR)), and *n* ^+^-*n*-*p*-*p* ^+^ (DDR) GaAs IMPATT diodes at both 35 and 60 GHz frequencies by using a small-signal (S-S) simulation technique based on drift-diffusion model. Though the S-S simulation provides substantial insight into the IMPATT operation, it has some intrinsic restrictions due to a number of unrealistic assumptions. Several important properties of IMPATT source admittance characteristics, RF power output, DC to RF conversion efficiency, and so forth, cannot be precisely determined from the S-S simulation. Thus L-S simulation is essential to acquire the above-mentioned properties accurately. Therefore in the present paper authors have used a nonsinusoidal voltage excited (NSVE) L-S simulation method developed by them [15–20] to investigate both the static (DC) and L-S characteristics of DDR IMPATTs based on 〈111〉, 〈100〉, and 〈110〉 oriented GaAs at different mm-wave atmospheric window frequencies, such as 35, 94, 140, and 220 GHz.

## 2. Large-Signal Modeling and Simulation Technique

**Figure 1:**
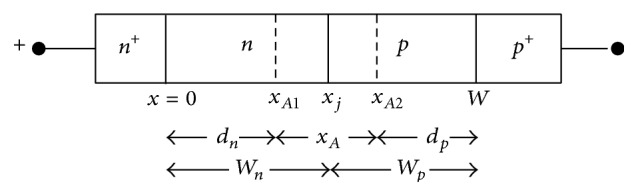
1-D model of DDR IMPATT diode.

Schematic of the one-dimensional (1-D) model of DDR IMPATT structure is shown in Figure 1. This 1-D model is used for the L-S simulation of the device. It is well known that the physical phenomena take place in the semiconductor bulk along the symmetry axis of the IMPATT devices. Thus the 1-D modeling and simulation of the device are absolutely justified. The fundamental time and space dependent device equations, that is, Poisson's equation, current continuity equations, and current density equations, are simultaneously solved under L-S condition subject to appropriate time varying boundary conditions by using a double-iterative simulation method [15–20] based on 1-D finite difference method (FDM). The fundamental device equations are given by

(1) Dxξx,t=qεsND−NA+px,t−nx,t,Dtp,nx,t =∓1qDxJp,nx,t+GAp,Anx,t+GTp,Tnx,t,Jp,nx,t=qp,nx,tvp,nx,t∓qDp,nDxp,nx,t,

where *D*_*x*_ and *D*_*t*_ are the partial derivatives with respect to *x* and *t*, respectively (*D*_*x*_ ≡ ∂/∂*x* and *D*_*t*_ ≡ ∂/∂*t*); all other symbols are carrying their usual significance. A list of symbols is given in appendix at the end of this paper where the usual meaning of each symbol is provided. The avalanche generation rates of both types of charge carriers at the space point *x* and at the time instant *t* are given by

(2) GAnx,t=GApx,t=nx,tαnx,tvnx,t +px,tαpx,tvpx,t.

The tunneling generation rate for electrons at the space point *x* at the instant *t* is a strong function of electric field at the same space point at the same instant. It can be derived from detailed quantum mechanical considerations [21–24]. In the present model, the authors have adopted Kane's model [22–24] of direct band to band tunneling assuming parabolic band approximation. It is given by

(3) $$\begin{array}{c} G_{Tn}\left(x,t\right)=a_{T}\xi^{2}\left(x,t\right)\exp\left(-\frac{b_{T}}{\xi \left(x,t\right)}\right). \end{array}$$

For parabolic band approximation the coefficients *a*_*T*_ and *b*_*T*_ can be expressed as [22]

(4) $$\begin{array}{c} a_{T}=\left(\frac{q^{2}}{8\pi^{3}\hbar^{2}}\right)\sqrt{\left(\frac{2m_{d}^{*}}{E_{g}}\right)},\\ b_{T}=\left(\frac{1}{2q\hbar }\right)\sqrt{\left(\frac{m_{d}^{*}E_{g}^{3}}{2}\right)}. \end{array}$$

**Figure 2:**
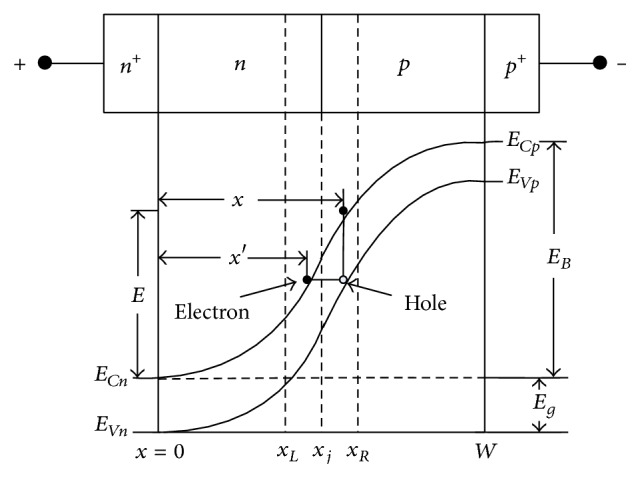
Energy-band diagram of reverse biased DDR IMPATT diode [24].

The energy-band diagram of reverse biased *n* ^+^-*n*-*p*-*p* ^+^ structure shown in Figure 2 is used to obtain the tunneling generation rate for holes. It is well known that the tunneling is an instantaneous phenomenon. The tunnel generation rate for holes at *x* at instant *t* is equal to that for electrons at some other space point *x*′ within the space charge layer at the same instant *t*. Thus

(5) $$\begin{array}{c} G_{Tp}\left(x,t\right)=G_{Tn}\left(x^{\prime },t\right), \end{array}$$

where the tunnel generation of an electron at *x*′ is simultaneously associated with the generation of a hole at *x*, where (*x* − *x*′) is the spatial separation between the edge of conduction band and valence band at the same energy. The relationship between *x* and *x*′ can be written as [23, 24]

(6) $$\begin{array}{c} x=\frac{x^{\prime }}{\sqrt{\left(1-E_{g}/E\right)}}\;\text{for}\;0\leq x\leq x_{j},\\ x=W-\left\{\frac{\left(W-x^{\prime }\right)}{\sqrt{\left[1+\left(E_{g}/E_{B}-E\right)\right]}}\right\}\;\text{for}\;x_{j}\leq x\leq W. \end{array}$$

The appropriate restrictions in (1) have been imposed via the time varying boundary conditions at the depletion layer edges. The boundary conditions for the time varying electric field at the depletion layer edges (i.e., at *x* = 0 and *x* = *W*) are given by

(7) $$\begin{array}{c} \xi \left(x=0,t\right)=\xi \left(x=W,t\right)=0. \end{array}$$

Similarly the boundary conditions for time varying normalized current density (*P*(*x*, *t*) = (*J*_*p*_(*x*, *t*) − *J*_*n*_(*x*, *t*))/*J*_*t*_(*t*)) at the depletion layer edges (i.e., at *x* = 0 and *x* = *W*) are given by

(8) $$\begin{array}{c} P\left(x=0,t\right)=\left\lfloor \left(\frac{2J_{p}\left(x=0,t\right)}{J_{t}\left(t\right)}\right)-1\right\rfloor ,\\ P\left(x=W,t\right)=\left[1-\left(\frac{2J_{n}\left(x=W,t\right)}{J_{t}\left(t\right)}\right)\right]. \end{array}$$

Time varying diode voltage (*V*_*t*_(*t*)) and time varying avalanche zone voltage drop (*V*_*a*_(*t*)) at any instant *t* can be obtained from the numerical integration of the field profile over the depletion layer and avalanche layer widths, respectively. Thus

(9) $$\begin{array}{c} V_{t}\left(t\right)=\overset{x=W}{\underset{x=0}{\int }}\xi \left(x,t\right)dx,\;\;V_{a}\left(t\right)=\overset{x=x_{A2}}{\underset{x=x_{A1}}{\int }}\xi \left(x,t\right)dx. \end{array}$$

DC values of the peak electric field (*ξ*_*P*_), breakdown voltage (*V*_*B*_), and avalanche zone voltage (*V*_*A*_) drop can be evaluated by taking the time averages of time varying peak electric field (*ξ*_*p*_(*t*)), diode voltage (*V*_*t*_(*t*)), and avalanche zone voltage (*V*_*a*_(*t*)) over a complete time period of steady-state oscillation (*T* = 1/*f*; where *f* is the fundamental frequency of steady-state oscillation). Thus the DC values of the peak electric field (*ξ*_*P*_), breakdown voltage (*V*_*B*_), and avalanche zone voltage (*V*_*A*_) are given by

(10) $$\begin{array}{c} \xi_{P}=\frac{1}{T}\overset{T}{\underset{0}{\int }}\xi_{p}\left(t\right)dt,\;\;V_{B}=\frac{1}{T}\overset{T}{\underset{0}{\int }}V_{t}\left(t\right)dt,\\ V_{A}=\frac{1}{T}\overset{T}{\underset{0}{\int }}V_{a}\left(t\right)dt. \end{array}$$

**Figure 3:**
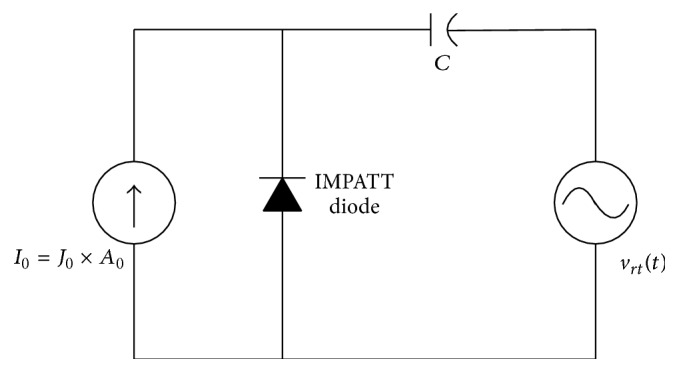
Voltage driven IMPATT diode oscillator and associated circuit.

In the present L-S model, the nonsinusoidal voltage excitation (NSVE) method is adopted. A nonsinusoidal voltage (*V*_RF_(*t*)) of following form,

(11) $$\begin{array}{c} v_{rf}\left(t\right)=V_{B}\overset{n}{\underset{p=1}{\sum }}{\left(m_{x}\right)}^{p}\sin\left(p\omega t\right), \end{array}$$

is assumed to be applied across the IMPATT device through a coupling capacitor (*C*) as shown in Figure 3. The fundamental frequency and number of harmonics of *v*_*rf*_(*t*) are *f* = *ω*/2*π* and (*n* − 1), respectively. The corresponding current response (*J*_*t*_(*t*) = *J*_0_ + *j*_*t*_(*t*); where *j*_*t*_(*t*) is the time varying current response of the device due to applied time varying voltage *r*_*rf*_(*t*) across it) of the device (which is biased with the DC current *I*_0_ = *J*_0_ × *A*_*j*_) is obtained from the L-S simulation. The voltage modulation factor (*m*_*x*_) is the measure of the amount of RF voltage swing over the DC breakdown voltage (*V*_*B*_) of the device. Thus the fundamental component of RF voltage may be written as *v*_*rf*1_(*t*) = *m*_*x*_ *V*_*B*_sin(*ωt*). The sufficient accuracy in the L-S simulation results can be achieved if the numbers of space and time stapes are taken within the range of 500–600 and 100–150, respectively.

Initially the L-S simulation is repeated for consecutive cycles to verify the stability of oscillation. After reaching steady-state, the time varying terminal current and voltage waveforms during a complete cycle of steady-state oscillation are Fourier transformed to obtain the frequency domain descriptions of those. Then the terminal current is divided by the terminal voltage (both are in frequency domain) to obtain the L-S admittance of the device (*Y*_*D*_(*f*)) as a function of frequency. The L-S admittance of the device is resolved into real and imaginary parts to obtain the L-S negative conductance (*G*(*f*)) and corresponding susceptance (*B*(*f*)) as functions of frequency (since *Y*_*D*_(*f*) = (*G*(*f*) + *jB*(*f*))*A*_*j*_; where *A*_*j*_ is the effective junction area of the device considering circular cross-sectional area of the device, that is, *A*_*j*_ = *π*(*D*_*j*_/2)^2^). Optimum frequency (*f*_*p*_) of the device may be obtained by finding out the frequency corresponding to the peak magnitude of negative conductance (|*G*_*p*_|) of the device. The L-S impedance of the device for any frequency *f* may be calculated from

(12) ZDf=1YDf=1Gf+jBfAj=ZRf+jZXf.

The L-S negative resistance (*Z*_*R*_(*f*)) and corresponding reactance (*Z*_*X*_(*f*)) of the device for any frequency *f* may be calculated from

(13) $$\begin{array}{c} Z_{R}\left(f\right)=\frac{G\left(f\right)}{\left\lfloor \left(G{\left(f\right)}^{2}+B{\left(f\right)}^{2}\right)A_{j}\right\rfloor },\\ Z_{X}\left(f\right)=\frac{-B\left(f\right)}{\left\lfloor \left(G{\left(f\right)}^{2}+B{\left(f\right)}^{2}\right)A_{j}\right\rfloor }. \end{array}$$

The RF power output can be calculated from the following expression:

(14) $$\begin{array}{c} P_{\text{RF}}=\left(\frac{1}{2}\right){\left(V_{\text{RF}}\right)}^{2}\left|G_{p}\right|A_{j}, \end{array}$$

where *V*_RF_ is the amplitude of fundamental component of the RF voltage. The L-S DC to RF conversion efficiency of IMPATT diode is given by

(15) $$\begin{array}{c} \eta_{L}=\left(\frac{P_{\text{RF}}}{P_{\text{DC}}}\right), \end{array}$$

where *P*_DC_ = *J*_0_ *V*_*B*_ *A*_*j*_ is the input DC power and *J*_0_ is the DC bias current density.

## 3. Design and Material Parameters

The widths of the *n*- and *p*-epitaxial layers (*W*_*n*_, *W*_*p*_), corresponding doping concentrations (*N*_*D*_, *N*_*A*_), and bias current density parameter (*J*_0_) of DDR IMPATTs based on 〈111〉, 〈100〉, and 〈110〉 oriented GaAs are chosen appropriately subject to the optimum performance of the device at different mm-wave frequencies (*f*_*d*_) by using the transit time formula by Sze and Ryder [25] and NSVE L-S simulation method [15–20]. The doping concentrations of *n* ^+^- and *p* ^+^-layers (*N*_*n*^+^_ and *N*_*p*^+^_) are taken to be much higher (~10^25^ m^−3^) as compared to those of *n*- and *p*-layers (*N*_*D*_ and *N*_*A*_). Structural and doping parameters of the devices are given in Table 1. Effective junction diameter (*D*_*j*_) is scaled down from 55 *μ*m to 20 *μ*m as the frequency of operation increases from 35 GHz to 220 GHz through a thorough steady-state thermal analysis for continuous-wave (CW) mode of operation, considering proper heat sinking aspects which is sufficient to avoid the thermal runway and burn out of the device [19] and corresponding *D*_*j*_ values are given in Table 1.

The electron (*a*_*n*_, *b*_*n*_) and hole (*a*_*p*_, *b*_*p*_) ionization coefficients in GaAs for different crystal orientations measured by Pearsall et al. [13] for the electric field range of 3.0 × 10^7^ to 6.6 × 10^7^ V m^−1^ are given in Table 2. The electric field dependence of *α*_*n*_ and *α*_*p*_ can be represented by the empirical relation [26] given by

(16) $$\begin{array}{c} \alpha_{n}\left(x,t\right)=a_{n}\exp{\left(-\frac{b_{n}}{\xi \left(x,t\right)}\right)}^{m_{n}},\\ \alpha_{p}\left(x,t\right)=a_{p}\exp{\left(-\frac{b_{p}}{\xi \left(x,t\right)}\right)}^{m_{p}}. \end{array}$$

The values of the constants *m*_*n*_ and *m*_*p*_ in the above equations are also given in Table 2.

The realistic electric field dependence of drift velocities (*v*_*n*,*p*_) of charge carriers and other material parameters such as bandgap (*E*_*g*_), intrinsic carrier concentration (*n*_*i*_), effective density of states of conduction and valance bands (*N*_*c*,*v*_), effective mass of electrons in conduction band (*m*_n_^*^) and that of holes in valance band (*m*_p_^*^), density of state effective mass of charge carriers (*m*_d_^*^), electron and hole mobilities (*μ*_*n*,*p*_), and diffusion lengths (*L*_*n*,*p*_) of 〈111〉, 〈100〉, and 〈110〉 oriented GaAs (at realistic junction temperature of 500 K) are taken from the published experimental reports [27].

## 4. Results and Discussion

The static or DC characteristics of the devices under consideration are obtained from the simulation by keeping the voltage modulation factor *m*_*x*_ = 0. The important DC parameters such as peak electric field (*ξ*_*P*_), breakdown voltage (*V*_*B*_), avalanche zone voltage drop (*V*_*A*_), ratio of drift region voltage drop to break down voltage (*V*_*D*_/*V*_*B*_; where *V*_*D*_ = *V*_*B*_ − *V*_*A*_), avalanche zone width (*x*_*A*_), and ratio of avalanche zone width to total depletion layer width (*x*_*A*_/*W*; where *W* = *W*_*n*_ + *W*_*p*_) of DDR IMPATTs based on 〈111〉, 〈100〉, and 〈110〉 oriented GaAs designed to operate at different mm-wave frequencies are obtained from the DC simulation and given in Table 3.

**Figure 4:**
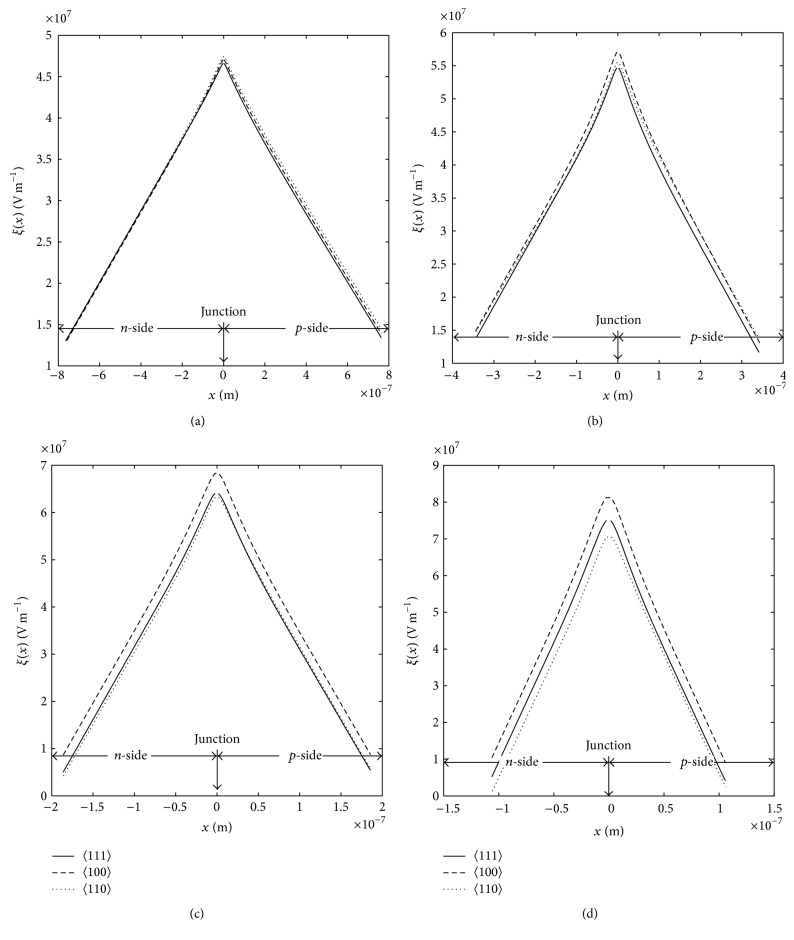
Static electric field profiles of (a) 35 GHz, (b) 94 GHz, (c) 140 GHz, and (d) 220 GHz DDR GaAs IMPATTs for different crystal orientation of GaAs.

The static electric field profiles of the above-mentioned devices are shown in Figures 4(a)–4(d). It is observed from Figures 4(a)–4(d) and Table 3 that peak electric field (*ξ*_*P*_) of the 35 GHz DDR diodes based on 〈110〉 oriented GaAs is highest among other diodes under consideration. However the same parameter of DDR diodes based on 〈100〉 oriented GaAs exceeds its other counterparts for higher mm-wave frequencies. The same nature is observed in breakdown voltage (*V*_*B*_); that is, the breakdown voltage is highest for the DDR diodes based of 〈100〉 oriented GaAs operating at 94, 140, and 220 GHz while at 35 GHz, the same is highest in 〈110〉 oriented GaAs based DDR diode. It is interesting to observe from Table 3 that the DDR diodes based on 〈111〉 oriented GaAs have the narrowest avalanche zone width (*x*_*A*_) and consequently minimum avalanche zone voltage drop (*V*_*A*_) up to 94 GHz, whereas, for the higher mm-wave frequencies, 〈110〉 oriented GaAs based DDR diodes possess the narrowest *x*_*A*_ and consequently minimum *V*_*A*_.

**Figure 5:**
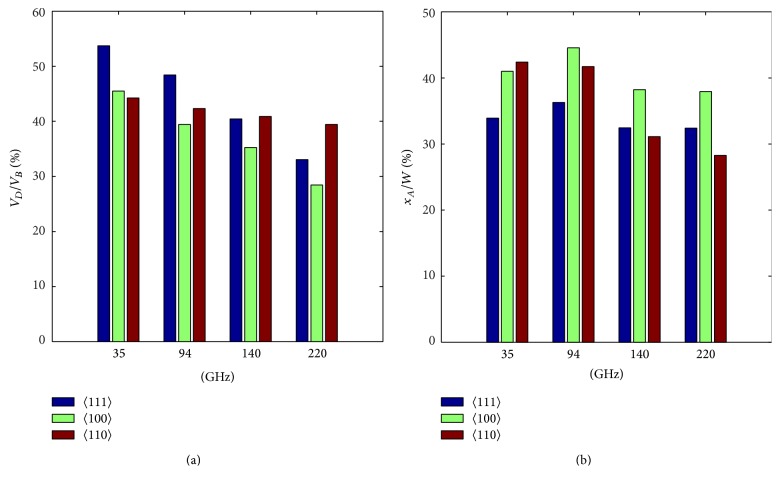
Bar graphs showing the (a) ration of drift zone voltage drop to breakdown voltage and (b) ration of avalanche zone width to total epitaxial layer width of 35, 94, 140, and 220 GHz DDR IMPATTs based on 〈111〉, 〈100〉, and 〈110〉 oriented GaAs.

Bar graphs in Figures 5(a) and 5(b) show the values of *V*_*D*_/*V*_*B*_ and *x*_*A*_/*W* (in percentage) for 35, 94, 140, and 220 GHz DDR IMPATTs based on 〈111〉, 〈100〉, and 〈110〉 oriented GaAs. Highest ratio of drift region voltage drop to breakdown voltage (*V*_*D*_/*V*_*B*_) and lowest ratio of avalanche zone width to total depletion layer width (*x*_*A*_/*W*) are observed in 〈111〉 oriented GaAs based DDR diodes up to 94 GHz, while the ratio of drift region voltage drop to breakdown voltage (*V*_*D*_/*V*_*B*_) and the ratio of avalanche zone width to total depletion layer width (*x*_*A*_/*W*) are found to be highest and lowest, respectively, in DDR diode based on 〈110〉 oriented GaAs at 140 and 220 GHz. Higher value of the ratio *V*_*D*_/*V*_*B*_ indicates greater DC to RF conversion efficiency (since *η*_*L*_ *∞V*_*D*_/*V*_*B*_) [28]. Therefore, *η*_*L*_ of DDR diodes based on 〈111〉 oriented GaAs are expected to be highest at 35 and 94 GHz, whereas at 140 GHz and 220 GHz the DDR diodes based on 〈110〉 oriented GaAs are expected to excel others at both 140 GHz and 220 GHz as regards the DC to RF conversion efficiency.

**Figure 6:**
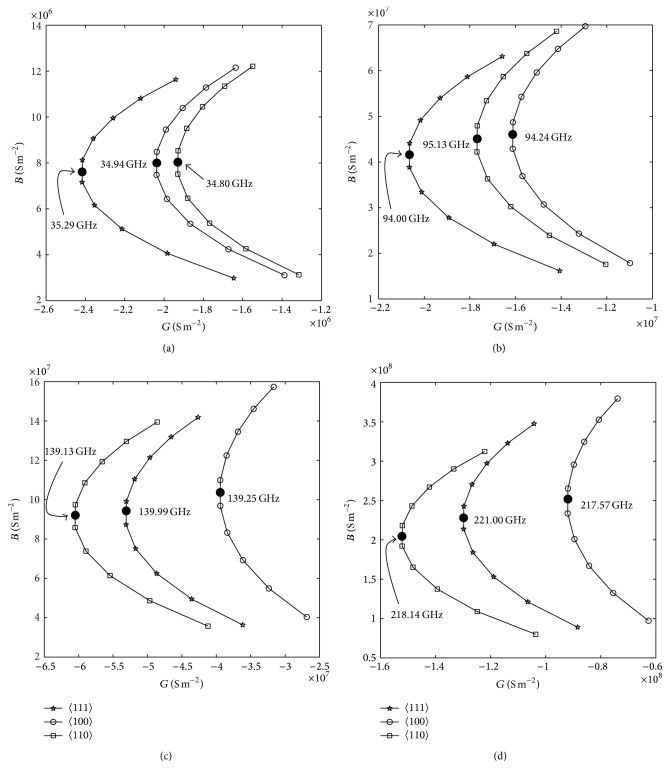
Admittance characteristics of (a) 35 GHz, (b) 94 GHz, (c) 140 GHz, and (d) 220 GHz DDR IMPATTs based on 〈111〉, 〈100〉, and 〈110〉 oriented GaAs.

The important L-S parameters such as optimum frequency (*f*_*p*_), avalanche resonance frequency (*f*_*a*_), peak negative conductance (*G*_*p*_), corresponding susceptance (*B*_*p*_), quality factor or *Q*-factor (*Q*_*p*_), negative resistance (*Z*_*R*_), RF power output (*P*_RF_), and L-S DC to RF conversion efficiency (*η*_*L*_) of DDR IMPATTs based on 〈111〉, 〈100〉, and 〈110〉 oriented GaAs designed to operate at 35, 94, 140, and 220 GHz for bias current densities of 0.85 × 10^8^, 5.60 × 10^8^, 10.20 × 10^8^, and 22.45 × 10^8^ for 50% voltage modulation are given in Table 4. The L-S admittance characteristics of the above-mentioned devices are shown in Figures 6(a)–6(d). It is interesting to observe from Table 4 and Figures 6(a)–6(d) that the magnitude of *G*_*p*_ is highest in DDR diodes based on 〈111〉 oriented GaAs up to 94 GHz, but beyond that frequency the magnitude of the same parameter is highest in the DDR diodes based on 〈110〉 oriented GaAs. Similar nature is also observed for *Z*_*R*_. *Q*-factor (*Q*_*p*_ = −*B*_*p*_/*G*_*p*_) of the device determines the growth rate and stability of IMPATT oscillation. Lower *Q*-factor closer to one (i.e., *Q*_*p*_ ≈ 1) suggests higher oscillation growth rate and better stability. It is interesting to note that, at higher mm-wave frequencies (i.e., at 140 and 220 GHz), the *Q*-factors of DDR diodes based on 〈110〉 oriented GaAs are smallest among all the devices under consideration, while, at 35 and 94 GHz frequencies, DDR diodes based on 〈111〉 oriented GaAs possess better oscillation growth rate and stability due to their smaller *Q*-factors as compared to DDR diode based on 〈100〉 and 〈110〉 oriented GaAs.

**Figure 7:**
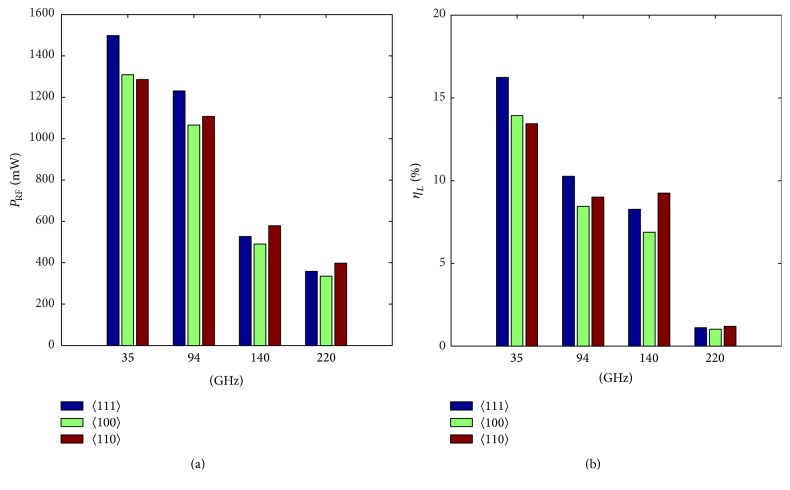
Bar graphs showing the (a) RF power output and (b) DC to RF conversion efficiency of 35, 94, 140, and 220 GHz DDR IMPATTs based on 〈111〉, 〈100〉, and 〈110〉 oriented GaAs.

Bar graphs in Figures 7(a) and 7(b) show the RF power output and DC to RF conversion efficiency of DDR diodes based on 〈111〉, 〈100〉, and 〈110〉 oriented GaAs designed to operate at 35, 94, 140, and 220 GHz frequencies. It is noteworthy from Table 4 and Figures 7(a) and 7(b) that DDR diodes based on 〈111〉 oriented GaAs are capable of delivering maximum peak RF power with maximum DC to RF conversion efficiency at 35 and 94 GHz. But at 140 and 220 GHz, both the *P*_RF_ and *η*_*L*_ are maximum in DDR diodes based on 〈110〉 oriented GaAs.

## 5. Conclusions

The L-S characteristics of DDR IMPATTs based on 〈111〉, 〈100〉, and 〈110〉 oriented GaAs designed to operate at mm-wave window frequencies such as 35, 94, 140, and 220 GHz are presented in this paper. Both the DC and L-S performance of the above-mentioned devices are investigated by using a NSVE L-S simulation technique developed by the authors. Results show that the DDR IMPATTs based on 〈111〉 oriented GaAs are most suitable for generation of RF power with maximum conversion efficiency up to 94 GHz. However, at higher mm-wave frequencies, the DDR IMPATTs based on 〈110〉 oriented GaAs exceed its other counterparts as regards both the RF power output and DC to RF conversion efficiency. Thus, for higher mm-wave frequencies greater than 94 GHz, 〈110〉 oriented GaAs substrate is best choice for the fabrication of DDR IMPATT device, while, up to 94 GHz, 〈111〉 oriented GaAs substrate must be preferred over other two orientations.

## Figures and Tables

**Table 1:** Structural, doping, and other parameters.

Design parameters	Design frequency, f_d_ (GHz)			
	35	94	140	220
*W*_*n*_ (*μ*m)	0.780	0.350	0.225	0.145
*W*_*p*_ (*μ*m)	0.800	0.350	0.225	0.145
*N*_*D*_ (×10^23^ m^−3^)	0.420	1.500	3.500	7.500
*N*_*A*_ (×10^23^ m^−3^)	0.400	1.500	3.400	7.500
*N*_*n*+_, *N*_*p*+_ (×10^25^ m^−3^)	1.000	1.000	1.000	1.000
D_j_ (*μ*m)	55.000	35.000	25.000	20.000

**Table 2:** Electron and hole ionization rate constants for 〈111〉, 〈100〉, and 〈110〉 oriented GaAs [13].

Carrier	*a*_*n*,*p*_ (×10^7^ m^−1^), *b*_*n*,*p*_ (×10^7^ V m^−1^) and *m*_*n*,*p*_	Crystal orientation		
		〈111〉	〈100〉	〈110〉

Electrons	*a*_*n*_	0.776	0.912	219.000
	*b*_*n*_	4.450	4.770	29.500
	*m*_*n*_	6.910	3.480	1.000

Holes	*a*_*p*_	63.100	34.700	34.700
	*b*_*p*_	23.100	21.800	22.700
	*m*_*p*_	1.000	1.000	1.000

**Table 3:** Static parameters of 35, 94, 140, and 220 GHz DDR IMPATTs based on 〈111〉, 〈100〉, and 〈110〉 oriented GaAs.

f_d_ (GHz)	Crystal orientation	J_0_ (×10^8^ A m^−2^)	ξ_P_ (×10^7^ V m^−1^)	V_B_ (V)	V_A_ (V)	V_D_/V_B_ (%)	x_A_ (*μ*m)	x_A_/W (%)
35	〈111〉	0.85	4.6622	45.69	21.14	53.73	0.536	33.92
	〈100〉	0.85	4.7048	46.51	25.35	45.49	0.648	41.01
	〈110〉	0.85	4.7423	47.37	26.41	44.25	0.670	42.41

94	〈111〉	5.60	5.4736	22.26	11.48	48.45	0.254	36.29
	〈100〉	5.60	5.7099	23.45	14.20	39.43	0.312	44.57
	〈110〉	5.60	5.5628	22.82	13.16	42.35	0.292	41.71

140	〈111〉	10.20	6.4306	12.71	7.57	40.44	0.146	32.44
	〈100〉	10.20	6.8431	14.24	9.22	35.27	0.172	38.22
	〈110〉	10.20	6.3306	12.28	7.26	43.32	0.140	31.11

220	〈111〉	22.45	7.5306	8.38	5.61	33.03	0.094	32.41
	〈100〉	22.45	8.1556	9.63	6.89	28.45	0.110	37.93
	〈110〉	22.45	7.0806	8.16	4.76	39.43	0.082	28.27

**Table 4:** L-S parameters of 35, 94, 140, and 220 GHz DDR IMPATTs based on 〈111〉, 〈100〉, and 〈110〉 oriented GaAs.

*f*_*d*_ (GHz)	Crystal orientation	*f*_*a*_ (GHz)	*f*_*p*_ (GHz)	*G*_*p*_ (×10^7^ S m^−2^)	*B*_*p*_ (×10^7^ S m^−2^)	*Q*_*p*_ (−*B*_*p*_/*G*_*p*_)	*Z*_*R*_ (×10^−9^ Ω m^2^)	*P*_RF_ (mW)	*η*_*L*_ (%)
35	〈111〉	22.91	35.29	−0.2417	0.7160	2.96	42.32	1498.50	16.24
	〈100〉	20.43	34.94	−0.2038	0.7475	3.67	33.95	1309.20	13.94
	〈110〉	19.92	34.80	−0.1930	0.7514	3.89	32.07	1286.10	13.44

94	〈111〉	70.08	94.00	−2.0650	3.8847	1.88	10.67	1230.60	10.26
	〈100〉	61.78	94.24	−1.6119	4.2898	2.66	7.68	1066.00	8.44
	〈110〉	64.88	95.13	−1.7686	4.2225	2.38	8.44	1107.60	9.01

140	〈111〉	107.50	139.99	−5.3110	8.7317	1.64	5.09	526.40	8.27
	〈100〉	94.00	139.25	−3.9431	9.6815	2.46	3.61	490.60	6.88
	〈110〉	107.91	139.13	−6.0521	8.5827	1.42	5.49	578.84	9.25

220	〈111〉	169.77	221.00	−12.9818	21.3811	1.65	2.08	358.00	1.11
	〈100〉	147.79	217.57	−9.2000	23.3634	2.54	1.46	335.00	1.02
	〈110〉	180.17	218.14	−15.2177	19.2157	1.26	2.53	397.90	1.19

## Appendix

### List of Symbols

*A*_*j*_: Effective junction area
*a*_*n*,*p*_: Ionization coefficients of electrons or holes
*α*_*n*_(*x*, *t*): Ionization rate of electrons at the space point *x* at time instant *t*
*α*_*p*_(*x*, *t*): Ionization rate of holes at the space point *x* at time instant *t*
*B*(*f*): Susceptance of the diode at frequency *f*
*b*_*n*,*p*_: Ionization coefficients of electrons or holes
*B*_*p*_: Susceptance of the diode corresponding to the optimum frequency *f*_*p*_
*D*_*j*_: Effective junction diameter
*D*_*n*,*p*_: Diffusion constant of electrons or holes
*E*: Measure of energy from the bottom of the conduction band on the *n*-side
*E*_*g*_: Bandgap of the semiconductor material
*ξ*_*P*_: DC peak electric field
*ξ*_*p*_(*t*): Time varying peak electric field
*ξ*(*x*, *t*): Electric field at the space point *x* at time instant *t*
*ε*_*s*_: Permittivity of the semiconductor material
*f*: Frequency
*f*_*a*_: Avalanche resonance frequency
*f*_*d*_: Design frequency
*f*_*p*_: Optimum frequency
*G*_*Ap*,*An*_(*x*, *t*): Avalanche generation rates of electrons or holes at the space point *x* at time instant *t*
*G*(*f*): Negative conductance of the diode at frequency *f*
*G*_*p*_: Peak negative conductance of the diode corresponding to the optimum frequency *f*_*p*_
*G*_*Tp*,*Tn*_(*x*, *t*): Tunneling generation rates of electrons or holes at the space point *x* at time instant *t*
*h*: Planck's constant (*h* = 6.625 × 10^−34^ J s)
*ħ*: Normalized Planck's constant (*ħ* = *h*/2*π*)
*J*_0_: DC bias current density
*J*_*n*_(*x*, *t*): Electron current density at the space point *x* at time instant *t*
*J*_*p*_(*x*, *t*): Hole current density at the space point *x* at time instant *t*
*J*_*t*_(*t*): Total terminal current
*j*_*t*_(*t*): AC component of the terminal current
*L*_*n*,*p*_: Diffusion lengths of electrons or holes
*m*_*d*_^*^: Density of state effective mass of charge carriers
*m*_*n*,*p*_: Constants associated with ionization rates of electrons or holes
*m*_*n*_^*^: Effective mass of electrons in conduction band
*m*_*p*_^*^: Effective mass of holes in valance band
*m*_*x*_: Voltage modulation factor
*μ*_*n*,*p*_: Mobility of electrons or holes
*n*_*i*_: Intrinsic carrier concentration
*N*_*A*_: Acceptor concentration of *p*-epitaxial layer
*N*_*c*_: Effective density of states of conduction band
*N*_*D*_: Donor concentration of *n*-epitaxial layer
*N*_*v*_: Effective density of states of valance band
*N*_*n*^+^_: Donor concentration of *n* ^+^-highly doped layer
*N*_*p*^+^_: Acceptor concentration of *p* ^+^-highly doped layer
*n*(*x*, *t*): Electron concentration at the space point *x* at the instant of time *t*
*η*_*L*_: DC to RF conversion efficiency
*P*_DC_: DC input power
*P*_RF_: RF power output
*p*(*x*, *t*): Hole concentration at the space point *x* at the instant of time *t*
*q*: Electric charge of an electron (*q* = 1.6 × 10^−19^ *C*)
*Q*_*p*_: Quality factor corresponding to the optimum frequency *f*_*p*_
*t*: Any time instant
*V*_*A*_: Avalanche zone voltage drop (DC)
*V*_*a*_(*t*): Time varying avalanche zone voltage drop
*V*_*B*_: Breakdown voltage
*V*_*D*_: Drift zone voltage drop (DC)
*V*_*t*_(*t*): Time varying diode voltage
*v*_*n*_(*x*, *t*): Drift velocity of electrons at the space point *x* at time instant *t*
*v*_*p*_(*x*, *t*): Drift velocity of holes at the space point *x* at time instant *t*
*v*_*rf*_(*t*): Nonsinusoidal RF voltage
*V*_RF_: Amplitude of the fundamental component of the RF voltage
*W*: Total depletion layer width (*W* = *W*_*n*_ + *W*_*p*_)
*W*_*n*_: *n*-side depletion layer width
*W*_*p*_: *p*-side depletion layer width
*ω*: Angular frequency
*x*: Any space point within depletion layer
*x*_*A*_: Avalanche zone width
*Y*_*D*_(*f*): L-S admittance of IMPATT diode for frequency *f*
*Z*_*D*_(*f*): L-S impedance of IMPATT diode for frequency *f*
*Z*_*R*_(*f*): L-S negative resistance of IMPATT diode for frequency *f*
*Z*_*X*_(*f*): L-S reactance of IMPATT diode for frequency *f*.
